# On the V-Line Radon Transform and Its Imaging Applications

**DOI:** 10.1155/2010/208179

**Published:** 2010-07-13

**Authors:** M. Morvidone, M. K. Nguyen, T. T. Truong, H. Zaidi

**Affiliations:** ^1^Laboratoire de Physique Théorique et Modélisation, CNRS UMR 8089, Université de Cergy-Pontoise, 2 av. Adolphe Chauvin, 95302 Cergy-Pontoise, France; ^2^Laboratoire Equipes de Traitement des Images et du Signal, CNRS UMR 8051/ENSEA, Université de Cergy-Pontoise, 2 av. Adolphe Chauvin, 95302 Cergy-Pontoise, France; ^3^Division of Nuclear Medicine, Geneva University Hospital, 1211 Geneva 4, Switzerland; ^4^Geneva Neuroscience Center, Geneva University, 1211 Geneva 4, Switzerland

## Abstract

Radon transforms defined on smooth curves are well known and extensively studied in the literature. In this paper, we consider a Radon transform defined on a discontinuous curve formed by a pair of half-lines forming the vertical letter V. If the classical two-dimensional Radon transform has served as a work horse for tomographic transmission and/or emission imaging, we show that this V-line Radon transform is the backbone of scattered radiation imaging in two dimensions. We establish its analytic inverse formula as well as a corresponding filtered back projection reconstruction procedure. These theoretical results allow the reconstruction of two-dimensional images from Compton scattered radiation collected on a one-dimensional collimated camera. We illustrate the working principles of this imaging modality by presenting numerical simulation results.

## 1. Introduction

Collecting first-order Compton scattered radiation by a two-dimensional gamma camera detection system from an object for three-dimensional imaging purposes has turned out to be an attractive alternative to conventional tomographic emission imaging, which operates only with primary (or unscattered) radiation [1, 2]. This new imaging principle is mathematically modeled by the so-called Conical Radon Transform (CRT) and has been supported by numerical simulations [2]. Later on, extensions of this idea have been advocated in various directions [3].

In this paper, we describe the implementation of this idea in two dimensions. Image formation is now modeled by a two-dimensional version of the CRT, which shall be called the V-line Radon transform. This imaging process may be applied, for example, to two-dimensional structures in material nondestructive testing as well as in biomedical imaging. Ideally, one can think of a flat object (or a material slice), which has been turned into an extended gamma ray-emitting object. This can be realized by injecting in its bulk medium a radiotracer which, after spreading unevenly throughout the body, emits gamma photons of primary energy *E*_0_.

However to an external gamma ray detector, such a uniform flat object does not appear “monochromatically” colored. The reason is that the emitted primary gamma photons will encounter electrically charged particles (electrons) within this object and will undergo Compton scattering. This scattering effect will render the object “polychromatically” colored or “white” because numerous scattered photons will be produced as secondary radiation over a wide range of energies below *E*_0_. In general, scattered radiation appears as noise or disturbances which degrades image quality in imaging units working with primary radiation. Thus removal (or at least drastic reduction) of the noise resulting from such physical degrading factors is an absolute requirement that remains an open research question.

In 2002, by reversing such traditionally admitted view, we have proposed a radically new standpoint [1]. It consists of collecting emanating scattered radiation over a whole range of scattered energies to build a new imaging principle. Data is collected by a nonmoving collimated linear gamma camera set to register an image at given scattered energy *E* < *E*_0_ (or equivalently at given scattering angle *ω*). Using an optical language, one may say that the gamma camera would record an image through a colored filter. The point is that the recorded images, labeled by the scattering angle *ω*, can be shown to constitute a complete set of data for image reconstruction. This is precisely what this V-line Radon transform is about.

Section 2 describes the image formation process by emission Compton scattered radiation and shows how the collected data by a linear collimated detector leads to a Radon transform of the object activity density on a pair of half-lines forming a standing letter V. This new integral transform, along with the conical Radon transform (CRT) [1–4], introduced a few years ago, becomes a new member of the rich family of Radon transforms [5], known so far in integral geometry as well as in tomographic imaging. Originally this V-line Radon transform has been proposed about a decade ago by Basko et al. [6] to model image formation in a two-dimensional Compton camera. However the Basko-Radon transform is defined in fact on a V-line with a swinging axis around a fixed site whereas the one considered here has a fixed axis direction. We study its properties and work out its kernel and its adjoint transform. In particular, we establish its analytic inverse and the corresponding filtered backprojection form. This last form has the advantage of reconstructing the image using fast algorithms. In Section 3, we present numerical simulations of image reconstruction including a thyroid phantom to support the feasibility of this imaging process and present related comments. The paper ends with a short conclusion on the obtained results and opens some future research perspectives.

## 2. The V-Line Radon Transformation

### 2.1. Image Formation and the V-Line Radon Transform

**Figure 1:**
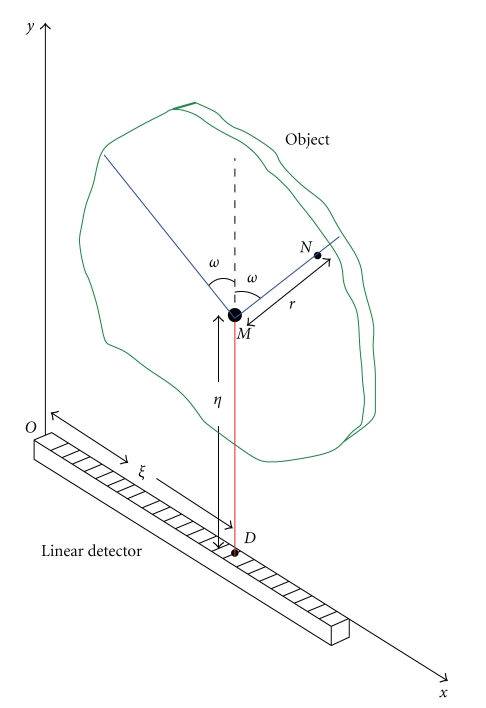
Experimental setup and definition of variables.

Consider a 2D object in which a nonuniform radioactivity source distribution exists and is represented by a nonnegative integrable function *f*(*x*, *y*) with bounded support. Figure 1 shows how a collimated linear detector is set parallel to the plane of the object and how it collects only outgoing radiation from the object which is parallel to the direction of the collimator holes.

When the detector is set to absorb gamma photons at energies below *E*_0_, the energy of primary photons emitted by the object, the photons have undergone at least one Compton scattering at a site **M** in the bulk of the object under a scattering angle *ω*. As the aim of the paper is to present a new imaging principle in 2D, we shall concentrate on the essence of the physical process and avoid, for the time being, addressing perturbing effects such as attenuation, higher-order scattering (of much lower actual occurrence probability), or any other interaction which may mask the proposed process.

The photon flux density measured at a detecting site **D** is due to the sum of scattered radiation flux densities outgoing from the set of scattering sites **M** lying along the axis of the collimator at **D**. As scattered photons have energy *E*, they have been deflected from an incident direction by a scattering angle *ω*, related to *E* by the Compton formula. Thus the totality of the detected flux density, for each scattering site **M**, is due to the sum of all point sources lying on the V-line with **M** as vertex. The analysis of this image formation can be formulated as follows.

Let *g*(*ξ*, *ω*) be the measured photon flux density at **D** under a scattering angle *ω*, using the cartesian coordinates of Figure 1. For ease of notation, we shall include all physical factors resulting from Compton scattering into one term *K*(*ω*). (This term contains the square of the classical electron radius, the average electron density, and the Klein-Nishina scattering probability function.) By computing the photon flux density with the two-dimensional photometric law, we can write *g*(*ξ*, *ω*) as a sum over all sites **M** = (*ξ*, *η*) of the V-line Radon transform:

(1) $$\begin{array}{c} g\left(\xi ,\omega \right)=K\left(\omega \right)\overset{\infty }{\underset{0}{\int }}\;\frac{d\eta }{\eta }\;T𝕍\;f\left(\xi ,\eta ,\omega \right), \end{array}$$

where

(2) $$\begin{array}{cc} T𝕍\;f\left(\xi ,\eta ,\omega \right) & =\overset{\infty }{\underset{0}{\int }}\;\frac{dr}{r}[f\left(\xi +r\sin\;\omega ,\eta +r\cos\;\;\omega \right)\\ & \;\;\;\;\;\;\;+f\left(\xi -r\sin\;\omega ,\eta +r\cos\;\;\omega \right)], \end{array}$$

the last integral is what we call the V-line Radon transform of *f*(*x*, *y*), because *f*(*x*, *y*) is integrated on a discontinuous vertically standing V-line. Thus we observe that image formation by first-order Compton scattered radiation in two dimensions leads to the new concept of a Radon transform on a V-line.

### 2.2. The *T* *𝕍* Transform

We examine a simplified case of the V-line Radon transform, for which the V-line vertex is on the *O* *x*-axis. This transform in fact models the imaging process of a collimated one-dimensional Compton camera, a special case of the Compton camera considered by Cree and Bones [7]. Primary radiation emitted from the object bulk is scattered by a linear scattering detector, which lies along the *O* *x*-axis of a cartesian coordinate system and is absorbed just on a next layer along the vertical direction by a second absorbing detector. This is of course a *ideal* hypothetical research camera, for which the V-line Radon transform models the image formation process.

The *T* *𝕍* transform of an activity density function *f*(*x*, *y*), defined as the integral of this function along a V-line, each branch of which making an angle *ω* with the vertical direction, gives the detected photon flux density:

(3) $$\begin{array}{cc} T𝕍f\left(\xi ,\omega \right) & =g\left(\xi ,\omega \right)\\ & =\overset{\infty }{\underset{0}{\int }}\;\frac{dr}{r}[f\left(\xi +r\sin\;\omega ,r\cos\;\;\omega \right)\\ & \;\;\;\;\;\;\;+f\left(\xi -r\sin\;\omega ,r\cos\;\;\omega \right)], \end{array}$$

for *ξ* ∈ ℝ and 0 ≤ *ω* < *π*/2.

*ξ* fixes the position of the vertex on the *O* *x*-axis. The factor 1/*r* in the integrand accounts for the photometric law of photon propagation in two dimensions (it would have been 1/*r* ^2^, in three dimensions). Here we have simplified the notations by absorbing the factor describing the Compton kinematics into the definition of *f*(*x*, *y*).

Equation (3) may be given another form with the following choice of variables *t* = tan*ω* and *z* = *r*cos  *ω*. Hence

(4) $$\begin{array}{c} g\left(\xi ,t\right)=\overset{\infty }{\underset{0}{\int }}\;\frac{dz}{z}\;\left[f\left(\xi +tz,z\right)+f\left(\xi -tz,z\right)\right]. \end{array}$$

It can be also put under the form of an integral transform, that is,

(5) $$\begin{array}{c} g\left(\xi ,\omega \right)=\int_{\mathbb{R}\times {\mathbb{R}}^{+}}dx\;dyk\left(x,y;\xi ,\omega \right)f\left(x,y\right). \end{array}$$

To obtain the kernel *k*(*x*, *y*; *ξ*, *ω*), we rewrite (3) as

(6) $$\begin{array}{cc} & g\left(\xi ,\omega \right)=\int_{\mathbb{R}\times {\mathbb{R}}^{+}}dx\;dyf\left(x,y\right),\\ & \overset{\infty }{\underset{0}{\int }}\frac{dr}{r}[\delta \left(\xi +r\sin\;\omega -x\right)Y\left(x-\xi \right)\\ & \;\;\;\;\;+\delta \left(\xi -r\sin\;\omega -x\right)Y\left(-x+\xi \right)]\delta \left(y-r\cos\;\;\omega \right). \end{array}$$

where *Y*(*x*) is the Heaviside unit step distribution. Since *y* > 0,  0 < *ω* < *π*/2, and tan *ω* > 0, the *r*-integration can be done with the variable *u* = *r*sin *ω*, that is,

(7) $$\begin{array}{cc} & \overset{\infty }{\underset{0}{\int }}\frac{du}{u}\;\delta \left(y-u\text{cot}\;\omega \right)[\delta \left(\xi -x-u\right)Y\left(x-\xi \right)\\ & \;\;\;\;\;\;\;\;\;+\delta \left(\xi -r\sin\;\omega -x\right)Y\left(-x+\xi \right)]\\ & \;\;=\frac{1}{\left(x-\xi \right)}\;\delta \left(y-\left(x-\xi \right)\text{cot}\;\omega \right)Y\left(x-\xi \right)\\ & \;\;\;+\frac{1}{\left(-x+\xi \right)}\;\delta \left(y-\left(-x+\xi \right)\text{cot}\;\omega \right)Y\left(-x+\xi \right). \end{array}$$

The kernel *k*(*x*, *y*; *ξ*, *ω*) can now be written under a compact form as

(8) $$\begin{array}{cc} k\left(x,y;\xi ,\omega \right) & =\frac{\cos\;\;\omega }{y}\;\delta \left(\cos\;\;\omega \left|x-\xi \right|-y\sin\;\omega \right)\\ & =\frac{\sin\;\omega }{\left|x-\xi \right|}\;\delta \left(y\sin\;\omega -\left|x-\xi \right|\cos\;\;\omega \right). \end{array}$$

### 2.3. The Inverse Transform *T* *𝕍* ^−1^

The inverse transform *T* *𝕍* ^−1^ can be worked out using Fourier transforms $\tilde{f}(q,y)$ (resp., $\tilde{g}(q,\omega )$) with respect to the variable *x* (resp., *ξ*) in *f*(*x*, *y*) (resp., *g*(*ξ*, *ω*)), that is,

(9) $$\begin{array}{c} g\left(\xi ,\omega \right)=\overset{\infty }{\underset{-\infty }{\int }}dq\;\tilde{g}\left(q,\omega \right)\exp\;\;\left(2i\pi q\xi \right),\\ f\left(x,y\right)=\overset{\infty }{\underset{-\infty }{\int }}dq\;\tilde{f}\left(q,y\right)\exp\;\;\left(2i\pi qx\right). \end{array}$$

Then (3) becomes

(10) $$\begin{array}{c} \tilde{g}\left(q,\omega \right)=\overset{\infty }{\underset{0}{\int }}\frac{dr}{r}\;\tilde{f}\left(q,r\cos\;\;\omega \right)2\cos\;\;\left(2\pi qr\sin\;\omega \right). \end{array}$$

After changing to variables *z* and *t* and defining $\tilde{G}(q,t)=\tilde{g}(q,\omega )$ with $\tilde{F}(q,z)=\tilde{f}(q,z)/z$, one finds that, after going to Fourier space, the V-line Radon transform appears as a Fourier-cosine transform

(11) $$\begin{array}{c} \tilde{G}\left(q,t\right)=\overset{\infty }{\underset{0}{\int }}dz\;\tilde{F}\left(q,z\right)2\cos\;\;\left(2\pi qzt\right). \end{array}$$

Let us point out that, for the conical Radon transform (CRT), passage to partial Fourier transform has led to a Hankel transform (or Fourier-Bessel transform) in which the kernel is a Bessel function *J*_0_ [8]. Here the role of the Bessel function *J*_0_ is played by a cosine function. Both transforms (Hankel and Fourier-cosine) are invertible transforms. Thus we can write down the inverse formula, thanks to the invertibility of the cosine transform:

(12) $$\begin{array}{c} \tilde{F}\left(q,z\right)=2\left|q\right|\;\overset{\infty }{\underset{0}{\int }}dt\cos\;\;\left(2\pi qtz\right)\tilde{G}\left(q,t\right). \end{array}$$

*f*(*x*, *y*) can be then reconstructed from its partial Fourier transform $\tilde{f}(q,y)=y\tilde{F}(q,y)$. A formula for the kernel of the inverse transform can now be derived as

(13) $$\begin{array}{c} k^{-1}\left(x,z\;|\;\xi ,t\right)=-\frac{z}{2\pi^{2}}\left[\frac{1}{{\left(x-\xi +zt\right)}^{2}}+\frac{1}{{\left(x-\xi -zt\right)}^{2}}\right]. \end{array}$$

This kernel is to be understood as a generalized function, or distribution and the corresponding integral as Cauchy principal value. Such a form is already known for the classical Radon transform [9].

### 2.4. The Adjoint Transform *T* *𝕍* ^†^

There is another formulation of the inversion procedure which lends itself more advantageously to algorithmic implementation. We call it * filtered backprojection*, due to its similarity to the standard Radon transform. In this section, we seek to construct the adjoint transform *T* *𝕍* ^†^ [10]. For all admissible pairs of functions (*f*(*x*, *y*), *g*(*ξ*, *ω*)) defined respectively in object space (*x*, *y*) ∈ ℝ^2^ and in image space (*ξ*, *ω*) ∈ ℝ × [0, *π*/2], we require that

(14) $$\begin{array}{c} \langle g\;|\;T𝕍\;f\rangle =\langle T{𝕍}^{†}g\;|\;f\rangle . \end{array}$$

Thus, the adjoint operator *T* *𝕍* ^†^ maps functions of variables (*ξ*, *ω*) onto functions of variable (*x*, *y*)

(15) $$\begin{array}{c} T{𝕍}^{†}:{ℋ}^{\prime }\left(\mathbb{R}\times \left[0,\frac{\pi }{2}\right)\right)\to ℋ\left(\mathbb{R}\times {\mathbb{R}}^{+}\right)\\ g\left(\xi ,\omega \right)\mapsto f\left(x,y\right) \end{array}$$

and takes the form

(16) $$\begin{array}{cc} T{𝕍}^{†}g\left(x,y\right) & =f\left(x,y\right)\\ & =\frac{1}{y}\overset{\pi /2}{\underset{0}{\int }}d\omega [g\left(x+y\tan\;\omega ,\omega \right)\\ & \;\;\;\;\;\;\;\;+g\left(x-y\tan\;\omega ,\omega \right)]. \end{array}$$

It can be checked that *T* *𝕍* ^†^ has the same kernel as *T* *𝕍*, see  (8),

(17) $$\begin{array}{c} k\left(\xi ,\omega ;x,y\right)=\frac{\sin\;\omega }{\left|x-\xi \right|}\delta \left(y\sin\;\omega -\left|x-\xi \right|\cos\;\;\omega \right). \end{array}$$

### 2.5. Filtered Backprojection Inversion Method

Let us recall that the most popular inversion method of the Radon transform is the so-called *filtered backprojection* method (FBP). This is an exact inversion formula obtained by combining the action of the ramp filter and the backprojection operation of the Radon Transform. In this section, we will demonstrate that the *T* *𝕍* transform may be inverted essentially in the same way, with the ramp filter and the backprojection operator associated to the *T* *𝕍* operator playing an analogous fundamental role.

Technically the backprojection principle consists in assigning the value *g*(*ξ*, *ω*) to every point on the “projection” V-line, which has given rise to this value, and then to sum over all contributions for every V-line “projection.” More precisely, we can say that the backprojection at angle *ω* in (*x*, *y*) is the sum of projections at angle *ω* at the points *ξ*_1_ = *x* + *y*tan *ω* and *ξ*_2_ = *x* − *y*tan *ω*, where (*x*, *y*) is projected:

(18) $$\begin{array}{cc} R_{\omega }\left(x,y\right) & =g\left(\xi_{1},\omega \right)+g\left(\xi_{2},\omega \right)\\ & =g\left(x+y\tan\;\omega ,\omega \right)+g\left(x-y\tan\;\omega ,\omega \right). \end{array}$$

The backprojection of every projection defines the backprojection operator *T* *𝕍* ^#^ which is obtained summing over every angle the expressions given in (18), and these are exactly the operations performed by the adjoint operator (16). The *y* factor appears because of the integration measure *d* *r*/*r* used in the definition of the projections (3).

Thus the backprojection operator is identical to the adjoint operator, that is,

(19) $$\begin{array}{c} T{𝕍}^{\#}g\left(x,y\right)=T{𝕍}^{†}g\left(x,y\right). \end{array}$$

Now the action of the ramp filter operator Λ over a function *f*(*x*, *y*) in the first variable is defined in the Fourier domain by

(20) $$\begin{array}{c} \tilde{\Lambda f}\left(q,y\right)=\left|q\right|\tilde{f}\left(q,y\right), \end{array}$$

where the Fourier transform is taken on the first variable *x*. From identity (12) we have

(21) $$\begin{array}{cc} f\left(x,y\right) & =2y\overset{\infty }{\underset{0}{\int }}dt\int_{R}dq\left|q\right|\tilde{g}\left(q,t\right)\cos\;\;\left(2\pi qty\right)e^{2i\pi qx}\\ & =y\overset{\infty }{\underset{0}{\int }}dt\int_{R}dq\left|q\right|\tilde{g}\left(q,t\right)e^{2i\pi qty}e^{2i\pi qx}\\ & \;+y\overset{\infty }{\underset{0}{\int }}dt\int_{R}dq\left|q\right|\tilde{g}\left(q,t\right)e^{-2i\pi qty}e^{2i\pi qx}\\ & =y\overset{\infty }{\underset{0}{\int }}dt\left(\Lambda g\right)\left(x+ty,t\right)+y\overset{\infty }{\underset{0}{\int }}dt\left(\Lambda g\right)\left(x-ty,t\right)\\ & =y\overset{\infty }{\underset{0}{\int }}dt\left[\left(\Lambda g\right)\left(x+ty,t\right)+\left(\Lambda g\right)\left(x-ty,t\right)\right]. \end{array}$$

In terms of the angle *ω*, the inversion formula reads

(22) $$\begin{array}{cc} \frac{f\left(x,y\right)}{y} & =\overset{\pi /2}{\underset{0}{\int }}\frac{d\omega }{{\cos\;\;}^{2}\omega }[\left(\Lambda g\right)\left(x+y\tan\;\omega ,\omega \right)\\ & \;\;\;\;\;\;\;\;\;\;+\left(\Lambda g\right)\left(x-y\tan\;\omega ,\omega \right)]. \end{array}$$

Defining the operator *ℳ*_*ω*_ as *ℳ*_*ω*_ *g*(*ξ*, *ω*) = *g*(*ξ*, *ω*)/cos ^2^ *ω* and having that

(23) $$\begin{array}{cc} T{𝕍}^{\#}g\left(x,y\right) & =\frac{1}{y}\overset{\pi /2}{\underset{0}{\int }}d\omega [g\left(x+y\tan\;\omega ,\omega \right)\\ & \;\;\;\;\;\;\;\;+g\left(x-y\tan\;\omega ,\omega \right)], \end{array}$$

we may write (22) as

(24) $$\begin{array}{c} \frac{f\left(x,y\right)}{y^{2}}=\left(T{𝕍}^{\#}{ℳ}_{\omega }\Lambda \;T𝕍f\right)\left(x,y\right). \end{array}$$

Finally, we recover the original density *f*(*x*, *y*) by a filtered-backprojection

(25) $$\begin{array}{c} f\left(x,y\right)=y^{2}\left(T{𝕍}^{\#}{ℳ}_{\omega }\Lambda \;T𝕍f\right)\left(x,y\right). \end{array}$$

This filtered backprojection inversion on V-lines is obtained for the first time. It generalizes the one known in the standard Radon transform on straight lines. The reconstruction formula (25) is mathematically equivalent to the reconstruction by *T* *𝕍* ^−1^ of the previous subsection. But the advantage of the filtered backprojection inversion formula is that it can be implemented by fast algorithms.

## 3. Numerical Simulations

**Figure 2:**
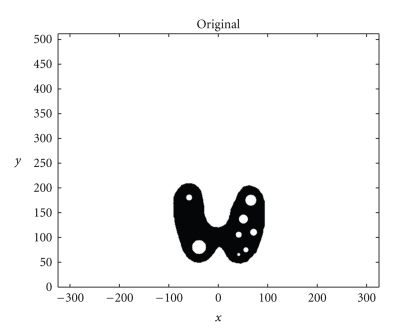
Original thyroid phantom.

**Figure 3:**
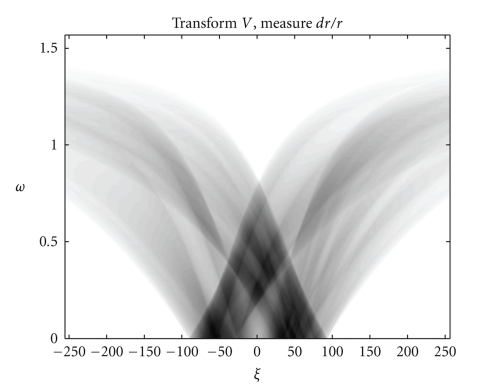
The *T* *𝕍* transform of the thyroid image shown in Figure 2 with *d* *ω* = 0.005 rad.

**Figure 4:**
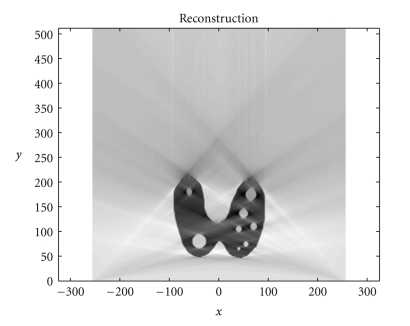
FBP-IM reconstruction of the thyroid phantom with *d* *ω* = 0.005 rad.

We present now the results of numerical simulations. The original image (Figure 2) of size 512 × 512 of length units is a thyroid phantom presenting with small nodules. Figure 3 shows the T*𝕍* transform of a thyroid phantom with angular sampling rate *d* *ω* = 0.005 rad and 314 projections (*π*/2/0.005 = 314) which are the images of Compton scattered radiation on the camera in terms of the distance *ξ* and the scattering angle *ω*. The reconstruction using FBP is given in Figure 4. The artifacts are due to the limited number of projections. Moreover, backprojection on V-lines generates more artifacts than backprojection on straight lines, because of more spurious line intersections. As our numerical results are based on the discretization of the inverse formula (22), a choice of a smaller discretization step *d* *ω* would improve image quality. This is indeed a well-established fact and in agreement with the improved sampling resulting from the increase of data collected at more values of the scattering angle *ω*. Despite these limitations, the small structures in the object are clearly reconstructed. This result illustrates undoubtedly the feasibility of the new imaging modality, for which the main advantage resides in the use of a * one-dimensional nonmoving* Compton camera for * two-dimensional* image processing. 

**Figure 5:**
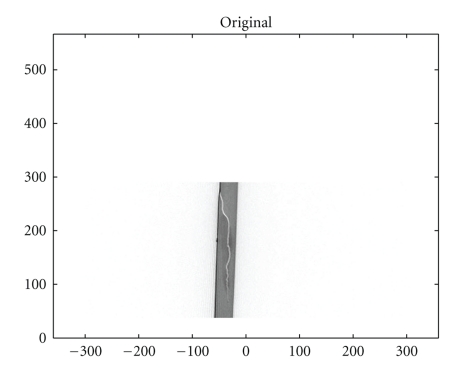
Original defect.

**Figure 6:**
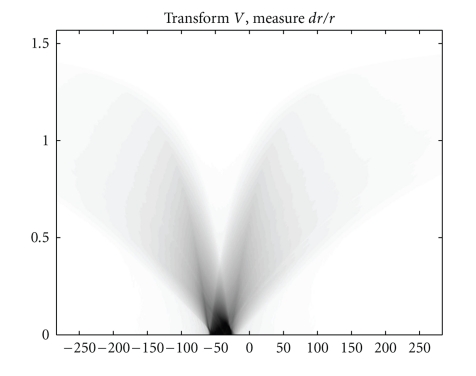
The *T* *𝕍* transform of the defect shown in Figure 5 with *d* *ω* = 0.005 rad.

**Figure 7:**
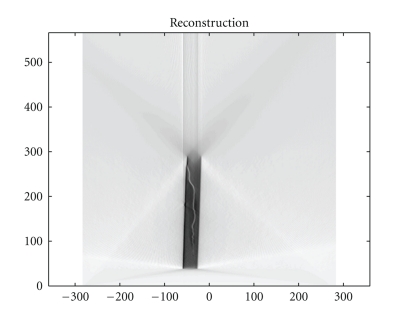
FBP-IM reconstruction of the defect with *d* *ω* = 0.005 rad.

We also present in Figures 5, 6, and 7 simulation results for a material defect under the same conditions to illustrate a possible application in industrial nondestructive control.

## 4. Conclusion

In this paper, a new class of Radon transform defined on a discontinuous line, having the shape of the letter V, is presented. We construct its analytic inverse transform as well as the corresponding filtered backprojection inversion method. The concept allows the two-dimensional image reconstruction from scattered radiation collected by a one-dimensional collimated camera. We have also performed numerical simulations to prove its practical viability. The obtained results provide stimuli for tackling the case of the swinging V-line Radon transform, for a two-dimensional Compton camera imaging, as proposed by [6]. Furthermore, the extension of this transform to a family of cones with swinging axis around a site in ℝ^3^, for a concrete gamma camera without mechanical collimator, poses a real mathematical challenge to overcome in the future.
